# How midwives and nurses experience implementing ten steps to successful breastfeeding: a qualitative case study in an Indonesian maternity care facility

**DOI:** 10.1186/s13006-022-00524-2

**Published:** 2022-12-03

**Authors:** Andini Pramono, Julie Smith, Siobhan Bourke, Jane Desborough

**Affiliations:** Department of Health Services Research and Policy, National Centre for Epidemiology and Population Health, Australian National University, Canberra, Australia

## Abstract

**Background:**

The in-hospital stay following childbirth is a critical time for education and support of new mothers to establish breastfeeding. The WHO/UNICEF ‘Ten Steps to Successful Breastfeeding (Ten Steps)’ was launched globally in 1989 to encourage maternity services to educate and support mothers to breastfeed. The strategy is effective, however its uptake within health systems and facilities has been disappointing. We aimed to understand midwives’ and nurses’ experiences of implementing the Ten Steps in an Indonesian hospital.

**Methods:**

This qualitative study was conducted in an Indonesian hospital which has been implementing the Ten Steps since the hospital’s establishment in 2012. Fourteen midwives and nurses participated in a focus group in January 2020. Data were analyzed using thematic analysis.

**Results:**

We identified five themes that represented midwives’ and nurses’ experiences of implementing the Ten Steps in this Indonesian maternity unit: 1) Human rights of child and mother, 2) Dependency on precarious leadership, 3) Lack of budget prioritization, 4) Fragmented and inconsistent implementation of the Ten Steps across the health system, and 5) Negotiating with family, community and culture. The results highlighted a dependency on local hospital champions and a lack of budget prioritization as barriers to implementation, as well as health system gaps which prevented the enablement of mothers and families to establish and maintain breastfeeding successfully in Indonesian maternity services.

**Conclusions:**

As Indonesia has one of the largest populations in South East Asia, it is an important market for infant milk formula, and health services are commonly targeted for marketing these products. This makes it especially important that the government invest strongly in Ten Steps implementation. Continuity of care within and across the health system and leadership continuity are key factors in reinforcing its implementation. The study findings from this Indonesian maternity care facility re-emphasize WHO recommendations to integrate the Ten Steps into national health systems and increase pre-service education on breastfeeding for health care professionals.

## Background

Breastfeeding is recognized internationally as the optimal infant feeding method, yet many women do not breastfeed for at least six months exclusively as recommended by health authorities [1]. The first few days of birth is critical, especially in developing the foundations for breastfeeding. New mothers need to adapt with their babies, learn their hunger cues, and at the same time recover from childbirth. Skilled and knowledgeable support for early initiation and establishment of breastfeeding from health facilities and health professionals providing maternity care is essential. The World Health Organization (WHO) has recommended that the Ten Steps to Successful Breastfeeding (hereafter referred to as the Ten Steps) be implemented in every maternity facility in order to ensure every mother receives adequate education and support for breastfeeding. The Ten Steps program was launched in 1989 [2], and developed into the Baby-Friendly Hospital Initiative (BFHI) in 1991 [3]. BFHI accreditation is awarded to maternity facilities that implement the Ten Steps and undergo external assessment and validation, with re-assessment required every three years [4, 5].

The Ten Steps and BFHI are key interventions that have been shown to be effective for increasing breastfeeding exclusivity and duration as well as child and maternal health, including in Low- and Middle-Income Countries (LMIC) and Asian country settings [6–10]. Nevertheless, the uptake within health system has been disappointing [11], with only 10% of births occur in BFHI accredited hospitals globally [12].

Breastfeeding is a human rights issue for mothers and babies [13]. In 2016 UN Human Rights Agencies reinforced women’s human rights on breastfeeding, including to be fully enabled to breastfeed such as by BFHI implementation, of adequate maternity care, and comprehensive implementation of the International Code of Marketing of Breastmilk Substitutes in all countries to protect informed decision-making. A recent review indicates that there is poor awareness of women’s human rights regarding breastfeeding in women’s sexual and reproductive rights literature, although children’s rights are better known [14].

The health importance of breastfeeding for children, including reduced risk of diabetes, ear infection, Sudden Infant Death Syndrome (SIDS) is well established [15, 16]. Breastfeeding has also been found to have short-term benefits for mothers, such as reducing the risk of postpartum hemorrhage by increasing the rate of uterine contraction, and also in the long term, such as reducing the risk of breast and ovarian cancer, type 2 diabetes and cardiovascular disease [17–20]. The economic impacts and costs of not breastfeeding are also increasingly evident. The global cost of not breastfeeding has been estimated at US$53.7 billion, based on future lost earnings due to premature child and maternal death as well as US$ 1.1 billion of healthcare treatment per annum [21].

There have been calls to invest more in breastfeeding policies and programs such as the BFHI for over two decades, including by UNICEF and WHO and the World Bank, and emerging research has focused on financing and economic aspects [22–25]. The positive social value of maintaining BFHI accreditation has been demonstrated in one hospital in Australia and in one hospital in Indonesia, which showed US$37 and US$49 benefit for every US$1 invested, respectively [26, 27]. Two other breastfeeding supporting programs also demonstrated positive social benefits [28, 29].

In Indonesia, the 1989 Ten Steps has been adopted by the Indonesian government since 1994 and regulated through *Peraturan Pemerintah No. 33 Tahun 2012* (Government Regulation No. 33 Year 2012 about Exclusive Breastfeeding) [30]; however, the country has not yet adopted the WHO’s updated 2018 Ten Steps [2]. Moreover, the Indonesian Ministry of Health had a ‘Mother and Baby Friendly Hospital’ (MBFH) program [31, 32]. Even though some of the 1989 Ten Steps were included in this program, it had a slightly different goal to the BFHI, aiming to decrease maternal and infant mortality rates, instead of focusing on breastfeeding success. Included in MBFH program were Step 1 (Have a written breastfeeding policy that is routinely communicated to staff) and Step 10 (Foster the establishment of breastfeeding support groups and refer mothers to them on discharge from the hospital) [32]. At the time of this study, the MBFH program was no longer in place.

**Fig. 1:**
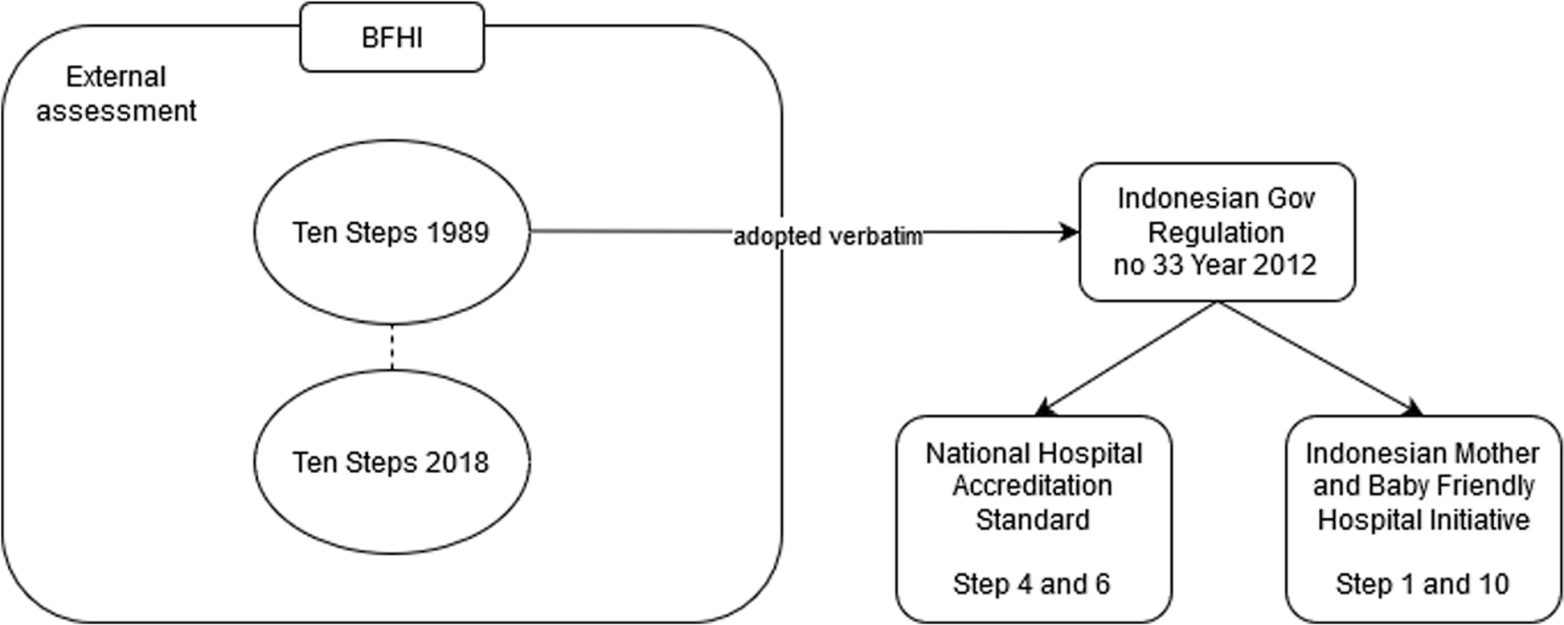
WHO/UNICEF BFHI/Ten Steps and its connection to Indonesian regulation (adopted from Pramono et al. [27])

Since 2018, Step 4 (Skin-to-skin contact) and Step 6 (exclusive breastfeeding in hospital) of 1989 Ten Steps have been integrated into Indonesia’s National Hospital Accreditation which was formulated and administered by *Komite Akreditasi Rumah Sakit* (KARS) or Hospital Accreditation Committee [33]. This accreditation program is compulsory for all hospitals in order to qualify for an operationalization license [33, 34]. The connection between these programs is described in Fig. 1.

Internationally, there is debate which distinguishes the effectiveness of Ten Steps implementation from the effectiveness of BFHI accreditation [35]. Ten Steps implementation alone is considered weak practice without external validation in place. In Indonesia, only 8% of government hospitals were categorized as implementing the Ten Steps in 2011 [36], and although 5.4% of hospitals in Indonesia were reported as BFHI accredited in an international report [12], there is no BFHI program at the moment in Indonesia. At hospital discharge, 62.7% of women are breastfeeding [36], but many infants have already been given formula which is known to reduce breastfeeding duration. In 2018, the exclusive breastfeeding rate in Indonesia was only 37.3% [37] as Indonesia is a large and rapidly growing market for milk formula products. Indonesian Ministry of Health has targeted 60% of babies under six months old exclusively breastfed by 2024 [38].

**Fig. 2:**
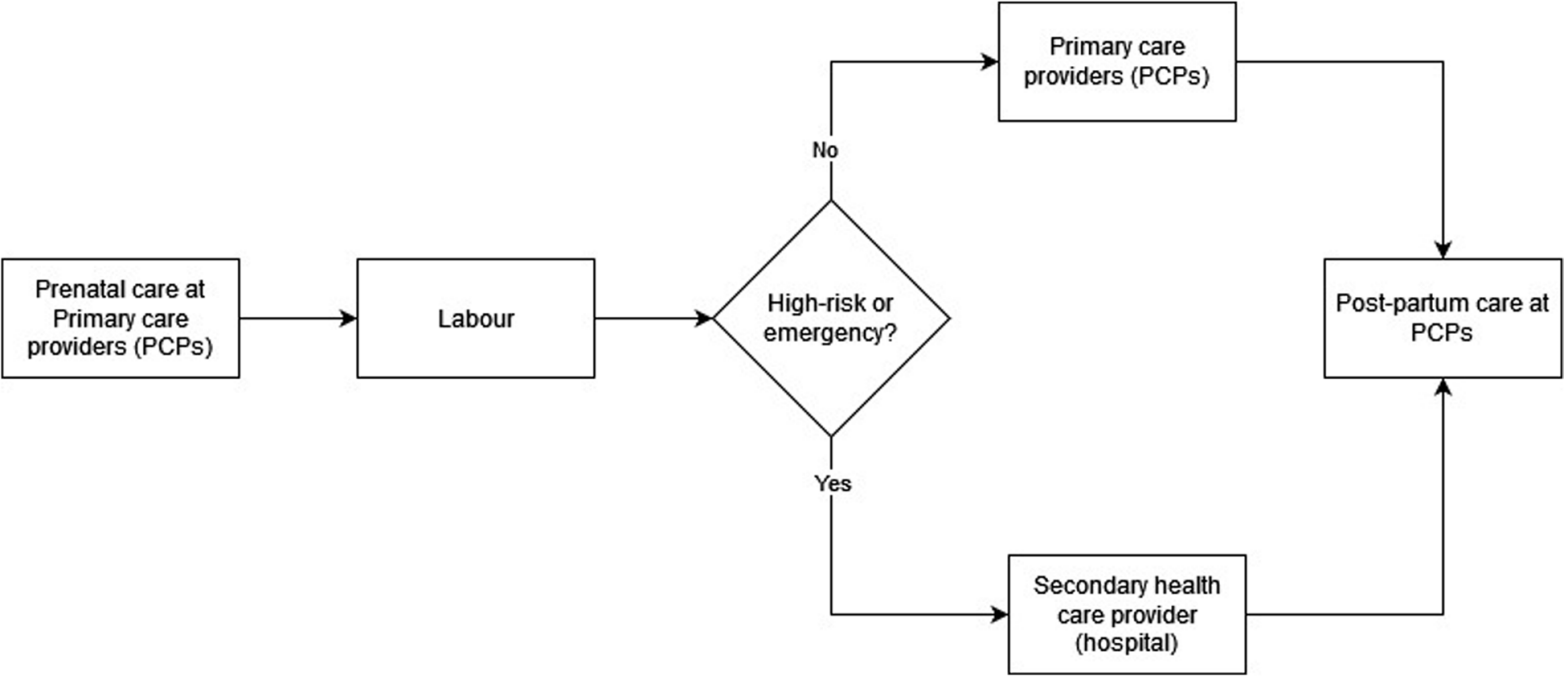
Maternity care service under Indonesian NHIS

In Indonesia, women can access free maternity facilities under the National Health Insurance Scheme (NHIS), administered by, and commonly known as, Badan Penyelenggara Jaminan Sosial (BPJS). Using NHIS, mothers receive antenatal care from GPs or midwives, although they cannot have the same health care professionals throughout pregnancy, labour and the post-partum period. Normal delivery services are carried out in primary health facilities and those with high-risk pregnancies are referred to secondary health facilities. Figure 2 describe the maternity care service under Indonesian NHIS.

The low breastfeeding rates in Indonesia are mostly due to lack of breastfeeding support in the health system and community, as well as widespread formula marketing, including through health professionals and services. The important role that midwives and nurses have in implementing the Ten Steps in maternity facilities is critical to the success of the Ten Steps; nevertheless there have been no studies of their experience with the Ten Steps as implemented in Indonesian hospitals. Therefore, we aimed to examine the perceptions and experiences of midwives and nurses regarding Ten Steps implementation in a hospital in Indonesia.

## Methods

### Study design

This study was part of a convergent parallel mixed-methods project that examined implementation of the BFHI in Australia and Ten Steps in Indonesia. The qualitative component was comprised of this exploratory qualitative study, conducted in Airlangga University Hospital (AUH) Surabaya, Indonesia, and counterpart qualitative study conducted in Calvary Public Hospital [39]. AUH was selected because AUH has implemented the Ten Steps since its establishment in 2012 and AP has network in this hospital. AUH is a teaching and referral hospital in Surabaya which is the second largest city in Indonesia and capital city of East Java Province with population of 2.9 million [40]. For the quantitative component, we measured the social return on investment (SROI) of the BFHI and Ten Steps implementation in these same Australian and Indonesian hospitals [26, 27]. Integration of the two components will be conducted using the non-adoption, abandonment, scale-up, spread and sustainability (NASSS) framework [41].

Ethical approval was obtained from the Human Research Ethics Committee (protocol number 2019/227) of the authors’ institute and the Airlangga University Hospital Ethical Committee of Research (number 162/KEP/2019).

### Recruitment

Participants were invited and provided information sheet by the Head of Midwifery Unit and Head of Nursing Services Unit. Participants were able to decline the invitation, however, no participant refused or dropped out of the focus group. Participants signed an informed written consent prior the focus group. The inclusion criteria were that they were a midwife or nurse with more than one year work experience in the birthing room, nursery room, pediatric clinic, operating theater, Maternal Neonatal Emergency Room (MNER), maternity ward, inpatient pediatric ward and Neonatal Intensive Care Unit (NICU). The sample was selected purposively to enable us to explore experiences and perspectives of midwives and nurses in all areas that have contact with birthing mothers. A total of 14 participants, consisting of six midwives and eight nurses, participated in the focus group. We took time to cover the topic as thoroughly as possible during the focus group which ensured that by the end of the discussion that no new information was emerging. We believe we reached data saturation Midwives had a minimum of three-year degree in midwifery, while nurses had minimum of three-year degree in nursing. Demographic data of the participants is described in Table 1.

**Table 1:** Focus Group Participants’ Demography

Characteristic	Number (*N* = 14)
Sex	
Male	2
Female	12
Profession	
Nurse	8
Midwife	6
Work area	
Birthing room	2
Maternity ward	2
Neonatal ICU (NICU) and Pediatric ICU (PICU)	2
Operating theater	1
Pediatric inpatient ward	1
Pediatric outpatient clinic	1
Nursery room	2
Maternal Neonatal Emergency Room (MNER)	1
all maternity and nursing unit	2
Breastfeeding counsellor	
Yes	3
No	11

### Data collection

The focus group was facilitated by AP with 14 participants on 20 January 2020 at AUH for one hour using Bahasa Indonesia. One research assistant who helped to organize and prepare the focus group also attended the focus group. It was audio recorded and then transcribed professionally. Participants’ confidentiality was protected by de-identifying all names in transcripts. Data was stored on a password protected computer at the university and only accessible to the primary researcher.

A semi-structured questionnaire was based on a previous study that examined the perceptions of South Australian midwives of the implementation of BFHI [42] and nine questions were added to explore the factors that were perceived as barrier and facilitator in the BFHI implementation (Table 2). This was used to explore participants’ experiences and perspectives of BFHI and Ten Steps implementation.

**Table 2:** Focus group questions

1	What is your opinion about the ideas behind the Baby Friendly Health Initiative?
2	What do you think about the Ten Steps to Successful Breastfeeding as a framework?
3	Why did your hospital decide to become BFHI accredited?
4	When you decided to become baby friendly what set of circumstances prevailed?
5	Who initiated the uptake of the baby friendly accreditation process in your hospital?
6	What made your baby friendly accreditation process successful?
7	Were there any barriers encountered to becoming BFHI accredited that were overcome?
8	How do you think baby friendly accreditation has affected maternity care specific to breastfeeding?
9	Are there any additional benefits of being BFHI accredited?
10	Are there any unexpected drawbacks of being BFHI accredited?
11	How much time does it take for each midwife to fulfil the requirements of the BFHI or Ten Steps?
12	How long does the training take?
13	Do you do the training in hospital time or your own time?
14	How often do you need to update?
15	Are there any costs associated with it?
16	Do you attend regular in-service education sessions about the BFHI or ten steps?
17	How difficult is it to implement these principles into practice?
18	What are some barriers to this?
19	What are some enablers in implementing it?

The research team was multidisciplinary and included clinical, academic and policy expertise. AP is an International Board-Certified Lactation Consultant (IBCLC) and a PhD candidate; JS is an Honorary Associate Professor, a qualified and experienced breastfeeding counsellor, and has expertise in breastfeeding economics, public regulation and policy, and gender analysis of policies; SB is a health economist and Research Fellow with expertise in economic evaluation and health services research, and JD is an experienced registered nurse and midwife, a Senior Research Fellow with expertise in qualitative research design and implementation. AP has a professional network in this hospital, however she was mindful not to share any personal or professional information with other team member. AP, JS and JD were aware of their perspectives towards breastfeeding and were careful be aware of any potential influence this might have on the data analysis.

### Data analysis

Audio recordings were transcribed and translated to English. Thematic analysis was conducted according to Braun and Clarke [43], and consisted of 1) data familiarization (all authors), 2) initial codes generations (all authors), 3) themes search (AP, JD), 4) themes review (AP, JS, JD), 5) themes naming and definitions (AP, JS, JD) and 6) report production (all authors). Phase 1 and 2 were conducted individually, before discussed as team. Between phase 2 and 3, AP input data to NVivo to assist analysis. Themes were all derived from data. We followed consolidated criteria for reporting qualitative research (COREQ) checklist.

## Results

Five themes were identified from the analysis: 1) Human rights of child and mother, 2) Dependency on precarious leadership, 3) Lack of budget prioritization, 4) Fragmented and inconsistent implementation of Ten Steps across the health system, and 5) Negotiating with family, community and culture.

### Theme 1: Human rights of child and mother

Participants emphasized their belief that, *“Humans should drink mother’s milk not cow’s milk to fulfil human rights*…” (Participant 2). This belief underpinned their everyday work in providing support and education to breastfeeding mothers. However, mothers’ rights to choose was also acknowledged “…*but it is their decision, depends on their families as well”*. Implementing the Ten Steps was participants’ passion, *“Since we're still young and idealist. We have passion to support patients.”* (Participant 11) with an underlying awareness of the implications of breastfeeding for future health and wellbeing, *“we shouldn't be selfish and should be aware of our children’s future”* (Participant 13)*.*

Participants also discussed their rights as employees to be supported to breastfeed *“We should also take care of the employees by providing facilities such as sofas, sink. So, we take care of patients, and we also get our rights.”* (Participant 7)*.* The facility has a lactation room that can also be used by staff, nevertheless “*We hope to have it in each floor”* (Participant 4).

### Theme 2: Dependency on precarious leadership

Participant admitted that *“The Ten Steps is implemented in this hospital since several years ago.—no infant formula or bottles, we also have a lactation room… In my division, the program runs well enough,—exclusive breastfeeding, breastfeeding counseling, breastfeeding techniques”* (Participant 4). At the same time, they understood how important the leaders were to maintaining commitment to the Ten Steps, *“It depends on the leader's vision and mission, whether he is profit-oriented or not. Sometimes people are too profit-oriented, then infant formula products marketing need to be increased to achieve targets. If the initial vision and mission is breastfeeding, and it is in the indicator, then the leader should stick to it. Don't let the baby formula enter the hospital. The procurement of government hospital will not be done if it’s not approved by the leader. If the leader is already supporting breastfeeding, the procurement will follow. But, if they are money-oriented, it would be dangerous.”* (Participant 12). Participants described the shared vision for breastfeeding that was currently in place at the hospital, “*…all teams in this hospital, the obstetrician and paediatrician also the surgeon have no problem with Early Initial Breastfeeding [EIB].”* (Participant 6). They proudly described their rooming-in unit. “*We also have a rooming-in unit for phototherapy. So, if the mother has been discharged or if the baby has been discharged and needs to do phototherapy, in the other hospital, the baby is isolated in the phototherapy room and the mother cannot stay. Here, we have a rooming-in unit.”* (Participant 11).

They expressed their concern when the leaders had to change. As a government hospital, leadership is assigned for five-year periods. *“Maybe since it's a government hospital, I mean, the executives and employees are always changing. So, when the executives are changed, we need to persuade them again to ascertain that it is Mother and Baby-Friendly Hospital. Because I know the offer from infant formula company is huge. So, I need to tell the executives that this hospital shouldn't accept that. And we don't know if there will be any change later. So, the obstacle is to maintain our vision. Employees keep changing, too. There are new staff. These new staff need to understand that this is pro-breastfeeding hospital.”* (Participant 11).

### Theme 3: Lack of budget prioritization

Despite having support from leaders, participants explained that there was no training specific related to breastfeeding for staff. *“…in training sessions, we don't only talk about breastfeeding, but all matters. And it's mainly related to SKP [Sasaran Kerja Pegawai/Employee Performance Target]. The breastfeeding is addressed together with skill competence. It can save the budget.”* (Participant 8). Participants compared their situation with hospitals that receive sponsorship from formula companies, and the perceived impact of not receiving this financial support was a lack of staff training, especially regarding breastfeeding. *“we don’t have enough budget. The budget for training will be allocated for other important necessities. There are other more important programs.”* (Participant 11). Comparing with their previous work experience, “*…We usually have many trainings, seminars. But, we don’t even have one.”* (Participant 10). This was perceived to be related to hospital leaders’ budgetary prioritization.

While midwives received breastfeeding education during their training degree in midwifery, nurses did not have that education *“…it will be better for nurses to get training.”* (Participant 12).

Lack of staff resources was described as one of the barriers in implementing Ten Steps. Especially in implementing Step 2, participants explained “*In the past, we had fewer patients. We could provide EIB around 1–2 h. Usually, we finished it in 2 h. But nowadays, the patients number increasing, so we can’t do that anymore.”* (Participant 8).

### Theme 4: Fragmented and inconsistent implementation of ten steps across the health system

Participants expressed their enthusiasm for the BFHI and regretted that there was no longer a specific accreditation. *“The accreditation standard for Mother and Baby-Friendly Hospital [MBFH] in Indonesia no longer exists, the last one was in 2007 or 2008. At the moment we don't have such specific accreditation. But the previous Hospital Accreditation Committee [Komite Akreditasi Rumah Sakit (KARS)] 2012 actually adopted the MBFH policies.”* (Participant 11).

Participants described their experiences when receiving patients who did not understand about Ten Steps implementation. “*If the patient has regular check up here they are aware that this hospital is pro-breastfeeding. But, most of our patients have never checked up here*.” (Participant 11). As part of the hospital’s standard procedures, patients were given a general information sheet and were required to provide consent prior to admission *“Because each patient should have been given general consent prior to admission and there are points regarding EIB and exclusive breastfeeding… But for emergency [caesarean] section patient—have they also been told during admission?..” That the patient should do EIB, how we do it. Or that this is Baby-Friendly Hospital, so the baby should be breastfed.* (Participant 8).

After discharge, mothers returned to the community health center to continue postnatal care. Participants were disappointed that they could not follow-up with mothers again. *“BPJS [Badan Penyelenggara Jaminan Sosial/Social Insurance Administration Organization] patients can’t do a check up here. That is the problem.”* (Participant 11).

### Theme 5: Negotiating with family, community and culture

Participants described challenges to enabling breastfeeding with family. *“In the birthing room, we provide early initiation of breastfeeding [EIB] to newborn baby. In the birthing room, the baby will stay for 2 h, including EIB for 1 h, and if the mother has recovered, we teach her how to breastfeed. This hospital supports them to learn how to breastfeed, but it is their decision, depends on their families as well. Often, the families are impatient. Such as, when the baby won't stop crying, they prefer to give the baby infant formula. At the end, it all comes back to their culture.”* (Participant 13).

Most participants agreed that family had strong influence, especially grandmothers. “*The complaints usually came from the grandmothers, "The baby is crying, don’t you care at all?"* (Participant 11). Participants believed that widespread formula marketing in the community had a great influence on family decisions, “*Some of them prefer formula*.” (Participant 10).

Mothers and families were also perceived to bring beliefs and traditions which sometimes were not beneficial in breastfeeding process, *“The colostrum was thrown away. We need to give them education. It’s because of the culture.”* (Participant 2) Participants agreed that this increased their workload as they needed to counsel and educate not only the mothers, but also grandmothers. “*We have difficulties to educate the family, especially the grandmothers.”* (Participant 9).

Participants admitted that *“if our patient is given infant formula by their family, we immediately tell them strongly, our voice intonation increase. And, if the patient is offered bottle filled with formula we'll immediately hold a counseling, how should I say it? Actually, it is not a problem, but it is better to tell them nicely. It is like we are accusing them. Although the intention is good”* (Participant 4). Participants felt that at times they were forcing the mothers to breastfeed, whereas it would be much more effective to educate them, so their decisions were informed and unforced, *“…when we told patients it seemed we've forced them like, "you should breastfeed the baby". Maybe we can educate them.* (Participant 8).

## Discussion

In this study we identified five themes that describe midwives and nurses’ experiences of implementing the Ten Steps in an Indonesian hospital. These were Human Rights of Child and Mother, Dependency on Precarious Leadership, Lack of Budget Prioritization, Fragmented and Inconsistent Implementation of Ten Steps across the Health System, and Negotiating with Family, Community and Culture. This study is the first qualitative study to examine midwives’ and nurses’ experiences of Ten Steps implementation in the context of national regulation on health system support for breastfeeding in Indonesia.

The first theme, of Human Rights of Child and Mother, reflected participants’ understanding that mothers have the right to make infant feeding decisions that are free from commercial influence [13]. As Indonesia is the biggest Muslim-majority country in the world [44], Indonesian mothers have strong beliefs that breastfeeding is also part of children’s basic natural rights. Breastfeeding babies until two years old or beyond is encouraged as part of Muslim religious, moral and spiritual understanding of breastfeeding as a basic natural right according to Quran, Muslim’s bible [45]. Nevertheless, in practice, breastfeeding is oftentimes challenged.

Breastfeeding benefits babies and mothers’ health regardless of their background, making it mothers’ and children’s right to be enabled to breastfeed. In the maternity care context this means having education and support for optimal infant feeding in order to make the best decision for their own and their child’s health [46]. As emphasized by UNICEF and WHO, breastfeeding has a critical role in addressing the Sustainable Development Goals (SDGs), especially improving child nutrition (SDG 2), preventing child mortality and decreasing the risk of non-communicable diseases (SDG 3) and supporting cognitive development and quality education (SDG4) [47].

Participants, many as parents themselves, understood that they also have rights to breastfeed while working, as recommended by International Labour Organization (ILO)’s Maternity Protection Convention, 2000 (No. 183) [48]. Research demonstrates that barriers to health staff breastfeeding at work include structural barriers, such as lack of space and time or flexibility to accommodate pumping, lack of maternity leave, and lactation policy to guide health staff who returned to work [49–51]. Sufficient paid parental leave is necessary to enable parents to continue breastfeeding and not force them to choose between their paid work and parenthood [46]. Challenges increased during the COVID-19 pandemic in which women health workers found it difficult to express their milk during work shifts as they had to wear layers of Personal Protective Equipment (PPE), and worried about the safety of the breastmilk [52], despite WHO guidance which encouraged breastfeeding continuation [53].

The second theme in this study was Dependency on Precarious Leadership; participants expressed their concerns regarding potential leadership changes and whether new leaders would have the same perspective regarding breastfeeding, specifically Ten Steps implementation. The challenge of implementing the Ten Steps has been reported in several studies and reports [54, 55]. Having support from hospital leaders and managers are among the facilitators [56, 57]. In Airlangga University Hospital (AUH), leaders were supportive and shared the same vision and mission to support breastfeeding programs. Therefore, they have implemented the Ten Steps since its establishment in 2012 and declared this publicly through the hospital director’s policy.

The third theme was Lack of Budget Prioritization. Evidence from our study also indicated that the cost of sustaining Ten Steps implementation was not considered in terms of financial planning, also reflecting experience elsewhere [12]. Having the Ten Steps implemented, especially Step 1, means that a hospital is not allowed to receive funding and/or sponsorship from infant formula manufacturers, which still commonly practiced in many hospitals [58–60]. It is widely known that infant formula manufacturers sponsors and/or fund healthcare professionals training, infrastructure development and/or medical equipment, as well as free or low-cost supply of formula [61]. Without external funding, hospital management needs to dedicate specific fund for breastfeeding education. In AUH, there was no regular staff training specific to breastfeeding as required in Step 2.

In addition to lack of breastfeeding training, participants discussed a lack of staff resources as one of the barriers to implementing Step 4 (early skin-to-skin contact). According to participants, this was because AUH is a referral hospital while the number of patients is increasing, midwife and nursing numbers are not increased to match. The mother-midwife/nurse ratio has been argued as one of the barriers to Ten Steps implementation in other countries [39, 62]. Furthermore, an Italian study showed that maternity units with lower midwife-infant ratios (1:2.5–1:5 vs 1:7–1:15) had higher exclusive breastfeeding rates [63]. Not including well babies in the ratio results in unrecognized workloads for midwives [64].

The fourth theme was Fragmented and Inconsistent Implementation of Ten Steps across the Health System. Midwives and nurses at AUH put more effort and time to educate mothers and families, as mothers and families did not believe they had received sufficient breastfeeding information during their antenatal checkups in community health centers. Continuity of care in the community health centers and hospital was considered one way to address this. Continuity of care is important in maternity care services [39, 65] as mothers who have good relationship with their midwives have better outcomes, including exclusive breastfeeding [66, 67].

As AUH is a referral hospital, many mothers have their antenatal check-up with their Primary Care Providers (PCPs). Mothers who give birth at AUH were referred by their PCPs; hence it was assumed that breastfeeding education during pregnancy was provided by their PCPs. Despite being regulated by the government, the Ten Steps implementation in PCPs has been found to be inadequate [68, 69]. This fragmented maternity care oftentimes hinders the breastfeeding success.

Participants discussed the fact that despite the inclusion of the MBFH program in the 2012 KARS program, it did not exist at the time of this study. Even though MBFH program targeted different goals to Ten Steps, they preferred and wished for integration of MBFH in the national accreditation program. Furthermore, the instrument of the national hospital accreditation was updated regularly, and participants perceived that the previous version of KARS instrument was more supportive to the Ten Steps. The integration of Ten Steps and BFHI in the national hospital accreditation program was also recommended by WHO in order to streamline and sustain the BFHI accreditation [12].

The last theme in this study was Negotiating with Family, Community and Culture. Another barrier in implementing Ten Steps in this Indonesian context was community belief and culture. Participants discussed how they had to negotiate with sometimes conflicting family preferences and culture about infant feeding and care. Misleading myths such as ‘colostrum is bad milk’ and ‘crying baby means not enough breastmilk is produced’ is common among grandmothers, who have significant influence on breastfeeding practice [70, 71]. Grandmothers’ complaints, as described by participants, further demonstrates how formula marketing exploits parents and families’ anxieties regarding their babies’ health. As well as marketing to health services and health professionals, industry marketing of formula milk products in the community adds to such misinformation, and is widespread in Indonesia. A recent WHO report demonstrates that the formula milk industry systematically undermines parents’ decision on infant feeding, compromising women’s and children’s health and human rights [61].

Participants in our study declared that they were passionate, idealist and dedicated to continue implementing the Ten Steps. Their understanding regarding breastfeeding as human rights also motivated them. Nevertheless, their dependency on the leadership and also inconsistency within the health system was a source of exasperation. Participants felt guilty about whether they advocated breastfeeding too strongly to mothers and families when there was insufficient education about breastfeeding in the community.

This study’s findings provide important evidence that health system and financing gaps prevent the enablement of mothers and families to successfully establish and maintain breastfeeding in maternity services in Indonesia. Serving large populations, the Indonesian government should ensure that the national policy and regulation on breastfeeding reaches the smallest unit of health care providers.

## Limitations

As this study was conducted in only one Indonesian hospital, there may be lack of generalizability. The findings might be similar for other hospitals in the Indonesian setting, but no guarantee for other country settings. There may also be participation bias in which midwives and nurses who took part in this study were those with strong belief in Ten Steps. Further exploratory research on health promotion of breastfeeding in Indonesian facilities drawing on Islamic teachings may also be helpful. While we interviewed midwives and nurses, understanding experiences from other health care professionals who have contact with pregnant and birthing mothers, would be beneficial to better understand and eliminate such barriers in implementing the Ten Steps. This study was conducted in a hospital that was implementing the 1989 Ten Steps, as the Indonesian government has not yet adopted the 2018 Ten Steps. This may limit the study findings, as the revised Ten Steps shift the focus from staff only education to the family enablement and education (for example Step 3 prenatal education, Step 8 infants’ feeding cues and Step 9 counselling for the use and risks of feeding bottles, teats and pacifiers), as well as continuous monitoring of the Ten Steps implementation. Hence, our findings on the women and families’ education and enablement may be questioned due to no requirement on the family education in the 1989 Ten Steps.

## Conclusion

As Indonesia has one of the largest populations in South East Asia, it is an important and rapidly growing market for infant milk formula, and health services are commonly targeted for marketing these products. Resistance to this marketing must emanate from a whole health service level rather from individual services. Implementation of the Ten Steps is highly dependent on the commitment of leaders, re-emphasizing the need to include the Ten Steps at the program level within health systems, thus ensuring that implementation is not dependent on individuals. Program level implementation may also lead to continuity of care across the Indonesian health system, another key factor for the success of the Ten Steps implementation. Our findings from this study in an Indonesian maternity care facility re-emphasize the WHO recommendations to integrate the Ten Steps into national health systems and increase pre-service education on breastfeeding for health care professionals.

## Data Availability

The primary data obtained through this component of the study was in the form of focus group audio recordings and transcripts. The dataset generated and analyzed during the current study is not publicly available. There are important ethical and confidentiality reasons why focus group data cannot be made open access, which would violate the terms of our ethical approval. We do not plan to provide direct access to the primary research data for these reasons, although additional data in the form of anonymized supporting quotes can be provided by the first author on reasonable request.

## Abbreviations

AUH: Airlangga University Hospital
BPJS: Badan Penyelenggara Jaminan Sosial (Social Insurance Administration Organization)
EIB: Early Initiation of Breastfeeding
KARS: Komite Akreditasi Rumah Sakit (Hospital Accreditation Committee)
